# Implications of Ramadan Fasting in the Setting of Gastrointestinal Disorders

**DOI:** 10.7759/cureus.36972

**Published:** 2023-03-31

**Authors:** Sedra Tibi, Saba Ahmed, Yasmeen Nizam, Murad Aldoghmi, Adam Moosa, Karim Bourenane, Mohsin Yakub, Hina Mohsin

**Affiliations:** 1 Medicine, California University of Science and Medicine, Colton, USA; 2 Medicine/Physiology, Nutrition, California University of Science and Medicine, Colton, USA

**Keywords:** diet education, ramadan fasting, liver disease, ibd, gerd, peptic ulcer disease, modifiable lifestyle behaviors, intermittent fasting, gastrointestinal disorder

## Abstract

Intermittent fasting is an increasingly popular dieting technique with many well-studied benefits, such as permitting weight loss in obese patients, lowering low-density lipoprotein cholesterol (LDL-C) levels and triglyceride levels, and optimizing circadian rhythms. A special type of intermittent fasting occurs during Ramadan, when Muslims worldwide fast daily from dawn to sunset for a month. Ramadan fasting has demonstrated several health benefits, including improving the gut microbiome, modifying gut hormone levels, and lowering proinflammatory markers such as cytokines and blood lipids. Although fasting has many health benefits, fasting during Ramadan may aggravate chronic medical conditions. We aim to review the literature devoted to Ramadan fasting and its effects on Muslim patients with gastrointestinal (GI) disorders, such as Inflammatory bowel disease (IBD), peptic ulcer disease (PUD), upper GI bleeding (UGIB), gastroesophageal reflux disease (GERD), and liver conditions. We will discuss recommendations for diet and medication compliance during Ramadan in the recommended pre-Ramadan counseling sessions. In this study, we used PubMed to research journals using the key terms "Ramadan," "intermittent fasting," and "gastrointestinal diseases." The current literature studying the impact of Ramadan on gastrointestinal disorders shows that patients with IBD have a minimal risk of disease exacerbation, although older men with ulcerative colitis (UC) were more prone to exacerbation during fasting. Patients with duodenal ulcers were at a higher risk of hemorrhage after Ramadan fasting. Although with mixed results, studies show patients with liver disease demonstrated improvements in liver enzymes, cholesterol, and bilirubin after Ramadan. Physicians should offer pre-Ramadan counseling to educate patients on the risks of fasting and encourage shared decision-making. To facilitate more definitive discussions between the physician and a Muslim patient, clinicians should seek a deeper understanding of how Ramadan fasting affects certain health conditions and offer accommodations, such as diet and medication adjustments.

## Introduction and background

Intermittent fasting has gained popularity over the past few years due to its overall positive effects on improving health, such as improving blood pressure, heart rate, cholesterol levels, and glycemic control [1]. There are various eating patterns for those who practice intermittent fasting, such as the 16/8 method, wherein individuals refrain from consuming any calories for 16 hours and have an 8-hour eating window for meals. Ramadan-associated intermittent fasting, hereafter referred to as "Ramadan fasting," is a special form of intermittent fasting practiced by 1.9 billion Muslims worldwide during the holy month of Ramadan, the ninth month of the Islamic lunar calendar. Muslims fast for 29-30 days, each day starting with a meal at dawn (suhoor) and breaking the fast with dinner at sunset (iftar). Between dawn and dusk, Muslims refrain from eating, drinking water, smoking, and having sexual intercourse. This religious obligation is taken very seriously by the vast majority of Muslims and has been practiced for over 1,400 years, as it is ordained in the Quran. "Believers! Fasting is enjoined upon you, as it was enjoined upon those before you, that you become God-fearing" [2]. Additionally, it is stated elsewhere in the Quran, "And eat and drink at night until you can discern the white streak of dawn against the blackness of the night, then complete your fasting until night sets in" [2].

There are some exceptions in regard to fasting for Muslims. These include prepubertal children, women during their menstrual period or postnatal bleeding, travelers, pregnant or breastfeeding women, the mentally unfit, and those with acute or chronic illness [3]. While there are several exemptions from fasting, many Muslim patients with acute or chronic medical conditions still choose to fast, which may adversely affect their health in certain conditions. Spirituality, religiosity, and personal beliefs are essential components of the social determinants that influence patients’ health behaviors and treatment adherence.

Since Ramadan fasting is a dietary modification, chronic medical conditions affected by dietary changes, such as diabetes and gastrointestinal (GI) diseases, may be exacerbated in Muslim patients with these chronic illnesses who are observing the fasts. This review article focuses on the effects of Ramadan fasting on the gastrointestinal system and in Muslim patients with certain gastrointestinal disorders, such as gastroesophageal reflux disease (GERD), inflammatory bowel disease (IBD), peptic ulcer disease (PUD), upper GI bleeding (UGIB), and liver conditions. By understanding the potential impact of Ramadan fasting on patients with chronic medical conditions, physicians can provide culturally competent care and better manage the care of their Muslim patients.

Methods

The protocol for this study was composed based on the Preferred Reporting Items for Systematic Reviews and Meta-Analyses (PRISMA) reporting guidelines. This review article of current and international literature was conducted using the major online database, PubMed. There was no start date restriction, and studies up to April 2022 were included. Studies and review articles were selected pertaining to the effect of Ramadan intermittent fasting on the body, particularly the gastrointestinal system, and its effect on gastrointestinal disorders such as inflammatory bowel disease, peptic ulcer disease, GERD, upper GI bleeding, and liver conditions. Key terms related to Ramadan fasting, gastrointestinal system effects, gastrointestinal disorders, gut microbiome, gut hormones, circadian rhythm, IBD, peptic ulcer disease, GERD, upper gastrointestinal hemorrhage, liver cirrhosis, liver disease, and diabetes were included in the searches. Additionally, for the topic of pre-Ramadan counseling, studies and review articles were selected on recommendations for diet and medication compliance during Ramadan fasting and general recommendations for Muslim patients observing Ramadan. Key terms related to Ramadan and diet modification, drug management, and GERD-specific recommendations were included in the search.

**Figure 1:**
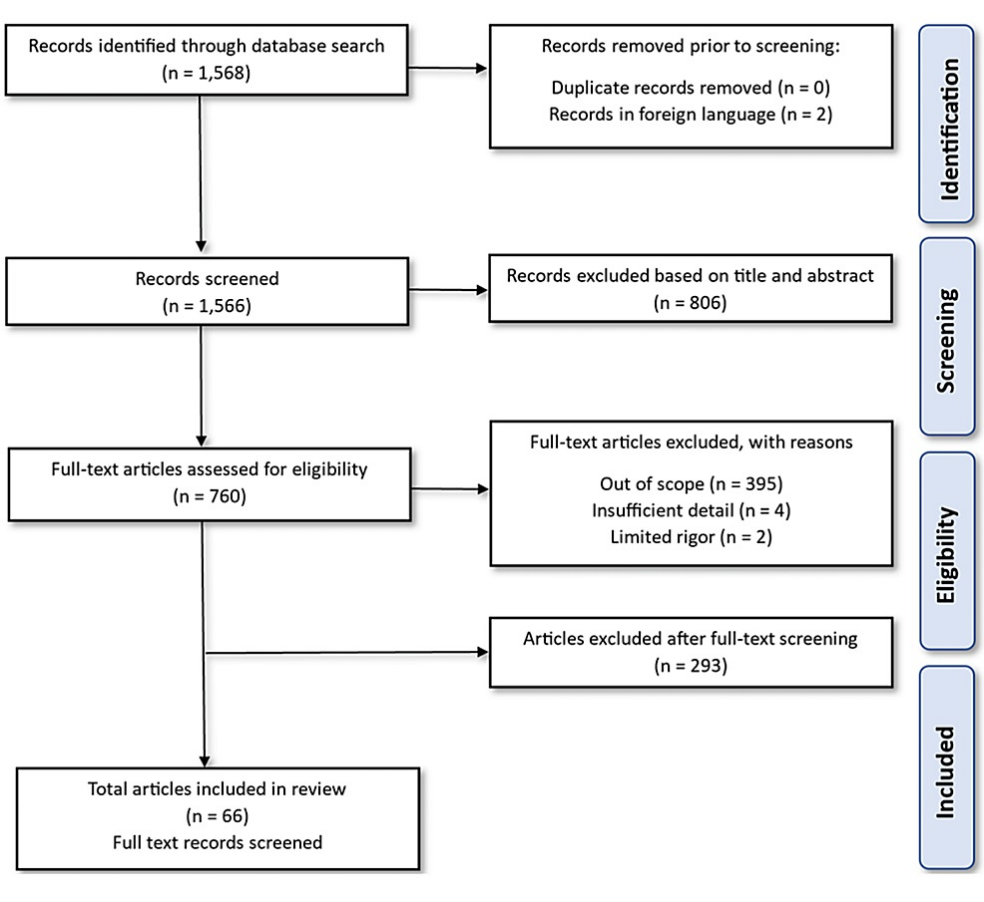
PRISMA flow diagram for selection of articles PRISMA: Preferred Reporting Items for Systematic Reviews and Meta-Analyses

Inclusion criteria included full-text articles in the English language that involved randomized control trials (RCTs) conducted on human subjects and systematic reviews evaluating the effect of Ramadan fasting on the gastrointestinal system, various gastrointestinal disorders, and diet and drug management during Ramadan. Exclusion criteria included studies that were not relevant to the research question, those that did not meet the inclusion criteria, and those with inadequate or incomplete data. A total of 1,568 papers were found on PubMed after searching the keywords "Ramadan fasting." After that, two papers were excluded because they were not in English, and 806 articles were excluded based on title and abstract. There was a selection of 760 full-text articles, out of which 395 were excluded for being out of scope, four were excluded for insufficient detail, and two articles were excluded for limited rigor. Additionally, 293 articles were excluded after the full-text screening. Finally, 66 full-text articles were selected that fulfilled the inclusion and exclusion criteria and were included in this systematic review. The screening for inclusion of articles in this systematic review is depicted in Figure 1. A standardized Microsoft Excel data collection form was used for data extraction, noting details related to the study characteristics, study design, variables under study, and a brief of the findings. Relevant literature was narrated in a concise thematic account, summarizing the key findings and highlighting any discrepancies or gaps in the literature.

## Review

Ramadan and general benefits

Ramadan is a specific type of fasting. It is a holy month observed by Muslims, who refrain from food, water, and sexual activity from sunrise to sunset for 30 days. This month is regarded as "a healthy non-pharmacologic means for minimizing risk factors (of various pro-inflammatory and atherosclerotic diseases) and improving health" [4]. With many patients observing Ramadan, it is critical that physicians understand the effects of Ramadan on the body and whether it should be recommended for individuals suffering from chronic conditions.

There are many reported health benefits attributed to observing Ramadan’s fast, notably changes in blood cellular components. According to an observational study performed in Lebanon that compared individuals observing Ramadan to those who did not, they found a general increase in dietary fiber intake in those observing the fast compared to non-fasters [5]. A review article by Wang and Wu [6] found that fasting generally results in an increase in lipolysis and free fatty acids and a decrease in blood lipids such as low-density lipoprotein cholesterol (LDL-C). Long-term fasting has been found to decrease HbA1c and blood glucose levels in diabetic patients [6]. The length of the fasting period affects the content, levels, and types of amino acids. A study conducted on GERD patients in Indonesia proposed that the possible explanation for low lipid blood profiles in prolonged fasts of over 10 to 12 hours is that the body scavenges other energy sources, including cholesterol and blood lipids [7].

A prospective observational study conducted in Iran on individuals who observed fasting and had cardiovascular risk factors found increases in high-density lipoprotein cholesterol (HDL-C), white blood cell count (WBC), red blood cell count (RBC), and platelet levels in those who fasted [8]. Decreased inflammation and pro-inflammatory cytokines such as IL-1b, IL-6, and TNFa were also observed [4].

Ramadan has a marked effect on physiological measures as well. A 2017 prospective controlled study conducted on young men in Germany found decreases in body weight, skeletal muscle mass, and free fat body mass [9]. Additionally, decreases in triglycerides, body mass index (BMI), and waist circumference have been reported in those who observed the Ramadan fast [8]. According to an observational study, systematic review, and meta-analysis performed in the UK, the researchers found that participants who fasted saw a reduction in systolic and diastolic blood pressure [10].

The practice of observing Ramadan fasting and a shift to nighttime eating is also accompanied by changes in sleeping and activity patterns, as well as circadian rhythm hormones, including cortisol, insulin, leptin, ghrelin, growth hormone, prolactin, sex hormones, and adiponectin [11]. A study conducted by Al-Rawi et al. [12] indicated that diurnal intermittent fasting during Ramadan significantly altered serum levels of ghrelin, melatonin, and leptin. At the end of Ramadan, serum levels of ghrelin, melatonin, and leptin significantly decreased, while salivary cortisol levels did not change compared to levels in the pre-fasting state. This can profoundly impact the circadian pattern of the body through significant metabolic changes. One particular study reported changes in the acrophase of proximal skin temperature (an indicator of core body temperature), indicating a shift delay in the circadian clock [13]. Growth hormone levels can also be altered during Ramadan. It is known to affect insulin sensitivity and act as an essential regulator of protein and fat metabolism and bone health. Secretion of growth hormone also follows a circadian pattern, with the highest peaks in the early morning. One study conducted by Ajabnoor et al. [14] reported that mean growth hormone concentrations during Ramadan were generally decreased. Moreover, the pattern of its secretion at the time of measurements changed, as the evening mean concentration was significantly lower than the morning mean only during Ramadan. This is most likely due to differences in sleeping and meal patterns and stimulation by food, and it is unlikely to contribute to the noted insulin resistance.

Ramadan and GI benefits

Intermittent fasting observed for one month during Ramadan is linked to various benefits for the gastrointestinal system. It was reported in one study conducted by Su et al. [15] that there is significant remodeling of the gut microbiome during Ramadan-associated intermittent fasting. In this study, the researchers analyzed the microbiome composition of 67 participants on days 1, 15, and 30 of Ramadan. Fecal samples from two cohorts (young male adults and middle-aged adults) in two different years were collected, and it was determined by 16S sequencing that those who underwent intermittent fasting had an increase in microbiome diversity, specifically the upregulation of the Clostridiales order-derived Lachnospiraceae and Ruminococcaceae bacterial families. This upregulation is correlated with the beneficial physiological effects of intermittent fasting, as Lachnospiraceae, together with other Clostridiales, are the primary source of butyrogenesis in the human intestine. Butyric acid is highly bioactive; for instance, it stimulates intestinal differentiation through the induction of Hedgehog signaling, and it promotes metabolic benefits via gut-brain neural circuits. Researchers have associated Lachnospiraceae species with improved health (reduced incidence of cancer, improved inflammatory bowel disease, better mental health, reduced atopy, and better cardiorespiratory fitness) [15]. Overall, this study suggests that intermittent fasting during Ramadan may be associated with favorable effects on the gastrointestinal system.

While it has been established that changes in diet affect the gut microbiome, how these changes manifest when fasting during Ramadan was investigated in a study by Ali et al. [16], focusing on three contributing factors: fasting, ethnicity, and dietary intake. Thirty-four healthy adult participants were grouped into Chinese and Pakistani ethnic groups and given questionnaires to assess their background, dietary habits, and antibiotic use. Researchers evaluated the effect of 29-day Ramadan fasting on alpha and beta diversity. Alpha diversity is the observed richness (number of taxa) or evenness (the relative abundances of those taxa) of an average sample within a habitat type. Beta diversity is the variability in community composition (the identity of taxa observed) among samples within a habitat [17]. For analysis, feces were collected before and after fasting. The results indicated that the population of some bacterial strains, such as Bacteroidetes and Firmicutes, increased in the Pakistani population following fasting. However, no noticeable changes were observed in the Chinese population. Additionally, they reported that fasting in both populations affected the beta diversity of the bacterial species. The results of most human studies indicate the positive effects of fasting on the composition and structure of the gut microbiome. Positive alterations in gut microbiota, such as overexpression of Akkermansia muciniphila, Bacteroides fragilis, Bacteroides, and butyric acid-producing Lachnospiraceae, were found to be associated with improved health indicators and decreased disease development during Ramadan fasting [18].

Moreover, one study conducted by Zouhal et al. [19] reported alterations in gut hormone concentrations in males with obesity in Tunisia following Ramadan-associated intermittent fasting. The principal finding is that Ramadan fasting has a beneficial effect on gut hormone levels for leptin, glucagon-like peptide-1 (GLP-1), peptide YY (PYY), and cholecystokinin (CCK) in males with obesity. Leptin concentrations increased following Ramadan fasting, suggesting a role in satiety, and GLP-1 concentrations decreased after Ramadan fasting. Leptin, a "satiety" hormone, is mainly produced by adipose tissue and the hypothalamus and inhibits hunger. GLP-1 stimulates insulin release from the pancreas, increases pancreatic beta cell volume, reduces glucagon release, and increases the feeling of fullness during and between meals by acting on the appetite centers in the brain and slowing the emptying of the stomach. The decrease in GLP-1 levels and a reduction in body fat percentage during Ramadan fasting also decrease the risk of obesity. The researchers proposed that the positive correlation between GLP-1 levels and body fat percentage observed may likely be due to reduced appetite following Ramadan fasting. Additionally, the study showed that PYY and CCK concentrations decreased significantly following Ramadan fasting. PYY is secreted into the blood by cells of the ileum and the colon following stimulation by nutrients from ingested food. Since food intake is reduced during the day in Ramadan fasting, this would consequently lower PYY and CCK levels. This suggests that not consuming food from dawn to dusk for 29-30 days affects these key hormones responsible for food intake and appetite control. In general, most studies indicate a favorable outcome following adherence to Ramadan fasting on the diversity of the intestinal microbiome and levels of gut hormones in combating obesity.

Another key player that enhances gut health during Ramadan-associated intermittent fasting is the migrating motor complex (MMC). MMC is a cyclic, recurring motility pattern that occurs in the stomach and small bowel during fasting and is interrupted by feeding. The MMC’s primary role in the digestive process is to clean out any undigested residual material in the gastrointestinal tract. Extra bile secretions are also observed during MMC, which is crucial in maintaining a healthy habitat for beneficial bacterial cultures. Essentially, the MMC acts as an intestinal house cleaner during the intermittent fasts in Ramadan. When the MMC is activated, smooth muscle contractions occur, during which stomach acid is secreted. The stomach acid will sweep away any residual undigested food particles and bacteria in the gut lining. Muscle contractions aid in moving particles into the pylorus, and the stomach contents move into the small intestine. Pancreatic and gallbladder enzymes are released to neutralize stomach acid, and then the small intestine moves the enzymes and stomach contents toward the colon. As bile moves through the gastrointestinal system, it starts killing off residual bacteria, preventing any from attaching to the gut wall. The bile is then redirected into the gallbladder and reabsorbed, during which antimicrobials are rereleased to eliminate any remaining bacteria. Bacteria and other stomach contents move to the colon until the next MMC [20]. Overall, fasting patterns during Ramadan can improve gastrointestinal motility associated with the MMC, allowing intestinal contents to move efficiently through the GI tract.

Additionally, fasting allows the gut to cleanse and strengthen its lining. It can stimulate autophagy, which helps the body recycle, remove degraded cellular components, and promote cellular renewal. During fasting, when the body is in a mild state of nutrient stress, the body’s cellular processes switch from a growth phase to a repair phase [21]. Fasting improves the process of elimination and increases the release of toxins from the colon, kidneys, bladder, sinuses, and skin. Essentially, restricting the incessant intake of foods for a month-long period during Ramadan allows the digestive system to rest, cleanse, and reset through MMC and autophagy. Ramadan fasting provides many health benefits to the gastrointestinal system by restructuring the gut microbiome, altering the levels of gut hormones, and essentially cleansing the gastrointestinal tract.

Ramadan fasting and gastrointestinal disorders

The systematic influences of Ramadan fasting are diverse, varying from alterations in lipid levels to modifications in hormonal secretion patterns. Moreover, several studies have analyzed the gastrointestinal-specific impacts of Ramadan fasting, which are similarly extensive, ranging from shifts in intestinal microbial composition to gut smooth muscle contractions. Since fasting can be considered a type of dietary adjustment, the possible outcomes of this food regimen in the context of diseased states are numerous. Patients with a high risk of severe health complications are discouraged from fasting. Therefore, data on the consequences of fasting in patients with certain conditions may provide a better understanding for physicians and Muslim patients to make informed health decisions. In the next section, we will review the available literature that has examined Ramadan fasting’s impact on specific gastrointestinal disorders and identify patterns to highlight the possible risks these patients should consider with fasting.

Inflammatory Bowel Disease

Inflammatory bowel diseases (IBD) incorporate a variety of disorders responsible for chronic inflammation of the gastrointestinal tract, leading to tissue damage, malabsorption, and systemic complications, with the two major categories being Crohn’s disease (CD) and ulcerative colitis (UC). Crohn’s disease is characterized by skip lesions and transmural (full thickness) inflammation of the intestinal mucosa involving any part of the GI tract with complications involving fistula, obstruction, nutritional deficiency due to malabsorption, and the risk for nephrocalcinosis [22]. Ulcerative colitis is defined by superficial and continuous colon ulceration with rectal complications such as toxic megacolon and a higher risk of colon cancer [22]. To minimize complications of IBD, patients are encouraged to avoid possible environmental exposures, dietary triggers, and psychological stress that may disrupt gut microbiota homeostasis, referred to as gut dysbiosis, which is believed to predispose patients to disease episodes [23,24]. Specific diets are known to alter gut microbiota and may play a role in changing the risk for symptom flares [25-27]. As mentioned above, Ramadan is considered a special diet that may cause gut microbiota dysbiosis and predispose individuals with IBD to flares or complications. Existing studies may shed light on the implications of IBD patients who choose to partake in Ramadan fasting, including the risks for new or worsened symptoms and disease severity.

Tavakkoli et al. [28] conducted a study on 60 patients with IBD (43 UC, 17 CD) in remission during 2006 who had no history of complications such as infection, perforation, or other comorbidities to assess their quality of life and possible links to Ramadan. These patients completed a self-conducted survey on symptoms before and after they fasted during Ramadan. They found that there was no statistically significant difference in their self-reported symptoms before and after Ramadan and no correlation between the number of days fasted and disease severity (which was based on self-reported symptoms). Their final recommendation was that Ramadan fasting posed no significant risks to patients with mild and uncomplicated IBD.

El Mountassir et al. [29] carried out a prospective study on 100 patients with CD (for an average age of 18 years) in 2011 to examine their clinical assessments of self-reported symptoms the day before Ramadan started (day 0), two weeks into fasting (day 14), and the last day of Ramadan (day 30). They observed that fasting was tolerated in 94% of cases, and the remaining 6% had interrupted fasting due to non-disease-related causes. Additionally, there were no reported negative symptoms associated with fasting in the findings of this study.

A prospective study by Negm et al. [30] recruited 80 patients (60 UC, 20 CD) who intended to observe Ramadan and had no comorbidities, history of perforation, or recent medication changes (within the last 12 weeks). These patients’ clinical state was assessed before and after Ramadan via serum CRP, stool calprotectin, and the Simple IBD Questionnaire (SIBDQ) to determine quality of life and clinical disease activity (partial Mayo score for UC, Harvey Bradshaw index (HBI) for CD). Two UC patients discontinued fasting on days 20 and 22 due to symptom flares that resolved with prednisone administration. The results for the remaining UC patients who completed fasting for the entirety of Ramadan showed a statistically significant increase in the partial Mayo score, notably in older men. A partial Mayo scoring index assessment is used for UC patients to determine if the individual is likely to be in remission, have mild disease, have moderate disease, or have severe disease based on self-reported bowel frequency, rectal bleeding, and a physician’s global assessment. Further findings showed no statistical significance in inflammatory markers (CRP, stool calprotectin), the HBI score in CD patients, or the SIBDQ score. Essentially, the study observed an increased risk of UC flares in elderly male patients and no changes in flare risk in patients with CD.

The number of available studies on Ramadan fasting and IBD is limited by small sample sizes, preventing a conclusive trend regarding the influence of fasting on Crohn’s disease or ulcerative colitis. Based on the available literature, Crohn’s disease patients with the mild and uncomplicated form of the disease have been observed to have a low risk for symptom flares associated with Ramadan fasting and demonstrated high tolerance levels. Patients with ulcerative colitis had variable outcomes. In two studies [28,29], patients with mild and uncomplicated disease had a low risk similar to CD, but the study conducted by Negm et al. [30] observed elderly UC patients having a higher risk of developing symptom flares and worse disease status compared to non-fasting patients.

The final recommendations offered by these studies include thoroughly counseling IBD patients on the possible risks of Ramadan fasting and allowing them to observe Ramadan with precautions and vigilance, with special attention on advising elderly UC patients.

Peptic Ulcer Disease

Peptic ulcers are characterized by disruptions in the mucosal layer of the gastrointestinal tract lining, commonly referred to as open sores or sloughing, and are mainly located in the stomach (gastric ulcer) or proximal duodenum (duodenal ulcer). These ulcers develop due to disruption of the protective mechanisms, notably mucus and bicarbonate, of the gastrointestinal mucosa, leaving it susceptible to damage by gastric acid and pepsin. The two most common etiologies are infection by Helicobacter pylori and long-term use of non-steroidal anti-inflammatory drugs (NSAIDs) or low-dose aspirin. Rising levels of antibiotic resistance and the increasing use of NSAIDs and aspirin for musculoskeletal and cardiovascular disease only contribute to the growing concerns about the development of PUD and its complications. Studies have suggested the possible role that diet plays in the risk of developing symptoms and complications of peptic ulcers. A cross-sectional study conducted in Japan revealed that certain foods, such as fruit juice and processed fish, were correlated with a decreased risk of H. pylori infection, and other foods were associated with a higher risk of atrophic gastritis [31]. This suggests an interplay between dietary intake and risk for peptic ulcer disease. The diet also adjusts gut microbial composition, a significant contributor to the infection and eradication of H. pylori. Based on evident correlations between peptic ulcer disease and dietary intake, Ramadan fasting may impact the symptoms and risk for complications of peptic ulcer disease.

Gokakin et al. [32] investigated 321 patients between 2009 and 2011 who underwent upper gastrointestinal endoscopy (UGE) due to indications of ulcer disease, mostly esophageal pain, dyspepsia, and anemia, with minimal differences in underlying comorbidities and indications of symptoms, and who observed Ramadan fasting. The findings of those who completed UGE the month before Ramadan (group 1), during Ramadan (group 2), and in the month after Ramadan (group 3) were compared. Based on the results of this study, there were no significant differences in the occurrence of gastritis, gastric ulcers, bile reflux, or gastric bleeding among these three groups. However, duodenal ulcers and duodenitis were detected significantly more frequently in group 2 than in groups 1 and 3. The authors recommended that patients with epigastric pain take precautions if they choose to fast, such as consulting their physician or taking antisecretory agents such as proton pump inhibitors (PPIs).

A retrospective study conducted by Chong in 2009 [33] similarly analyzed the findings of upper gastrointestinal endoscopy for 1,661 patients referred for dyspepsia the month before Ramadan, during Ramadan, and the month after Ramadan for four consecutive years (2004-2007). Their results demonstrated no statistical significance between the three months for gastric pathologies, including gastritis, gastric ulcers, or other significant gastric pathologies. However, there was a significant increase in duodenal findings during Ramadan, notably duodenitis and duodenal ulcers.

Malik et al. [34] conducted a study to compare upper endoscopies performed seven days prior to Ramadan and seven days after Ramadan in patients with endoscopically proven peptic ulcer disease. Patients from this study were prescribed H2-blocker drugs to be taken at suhoor (the dawn meal) and iftar (the evening meal) for the 23 fasting patients and 15 non-fasting controls. Patients who fasted and complied with treatment showed healing in active acute duodenal ulcers and erosive duodenitis. Patients with active chronic duodenal ulcers and chronic gastric ulcers had no signs of healing. Based on the findings of this study, chronic ulcers are likely difficult to heal during fasting. However, this study was performed with a small sample size of patients. Larger studies may shed more light on the impact of Ramadan fasting on the healing of chronic peptic ulcers.

Kocacusak [35] performed a retrospective study in Turkey, a Muslim-majority country, to compare the monthly rate of surgical interventions for peptic ulcer perforation between surgeries performed between 1979 and 2016 during non-Ramadan months, labeled Group 1 (1,805 patients over 396 months), and during Ramadan, referred to as group 2 (506 patients over 36 months). Their findings included a significant increase in the mean monthly number of surgical intervention rates for peptic ulcer perforation in group 2 (14.05 vs 4.55, p < 0.001). The findings also noted a statistically significant increase in the number of male patients compared to females in the months of Ramadan. The author proposes that this may be attributed to females not fasting during the days of their menstrual cycle, while male fasting is consecutive. There were no significant differences in the mortality rate between groups 1 and 2.

The available studies investigated the influence of Ramadan fasting on the symptoms, complications, and healing of gastric and duodenal ulcers. Essentially, the studies had findings in agreement with gastric ulcers having a low risk of symptom flares and impaired healing associated with Ramadan fasting. However, the study performed by Kocacusak [35] found an increase in surgical interventions required for peptic ulcer perforations in fasting months compared to non-fasting months. Regarding duodenal ulcers, studies have found that Ramadan fasting exacerbates duodenal ulcer symptoms and incidence. Theoretically, this may be attributed to the mechanism of food activating bicarbonate and mucus production in the small intestine and relieving duodenal ulcer pain [36].

In addition to counseling Muslim patients on the risks and benefits associated with Ramadan fasting, patients with duodenal ulcers should also be informed of the above findings. Within this discussion, emphasis should be placed on the importance of carefully monitoring one’s own symptoms and seeking medical attention if symptoms arise.

Gastroesophageal Reflux Disease

GERD is the retrograde flow of stomach contents that irritates the esophageal lining, leading to many symptoms of discomfort. Some common symptoms of GERD include retrosternal burning pain, regurgitation, chest pain, and dysphagia. The risk and aggravating factors of GERD include obesity, older age, use of analgesics, smoking, alcohol consumption, intake of caffeinated drinks, and psychological distress [37-39]. As Ramadan fasting is considered a diet, during Ramadan, some of these behaviors are changed for the better, including the cessation of smoking and drinking alcohol. With the reduction of these habits known to worsen GERD symptoms, there may be an alleviation of symptoms during Ramadan [7].

A longitudinal study conducted by Mardhiyah et al. [7] evaluated 130 people divided into a fasting group (66) and a non-fasting group (64) using a GERD questionnaire. The GERD-Q study evaluated GERD complaints and consisted of six questions asking about GERD symptoms assessed in the fourth (final) week of Ramadan and three months after. In this study, there was a significant difference in the median GERD-Q score between Ramadan-fasting patients and non-fasting group subjects in both the Ramadan and non-Ramadan fasting months, indicating that GERD symptoms were milder for those who fasted during Ramadan than for those who did not and that GERD symptoms were lessened in the month of Ramadan versus non-Ramadan months. This study tested whether smoking cessation was a confounding variable and found that when they compared the non-smoker subjects who fasted versus those who did not fast, there was still a significant difference between the two groups, showing that GERD complaints were milder during Ramadan fasting. This indicates that smoking cessation is not the only factor contributing to reduced GERD complaints. They concluded that GERD issues were less severe during Ramadan than during the non-Ramadan months.

Rahimi et al. [40] conducted a longitudinal study observing the symptoms of GERD during Ramadan involving 69 patients separated into two groups. The groups consisted of 33 patients in the fasting group and 36 in the non-fasting group. The time frame of this study consisted of three months: one month before Ramadan, one month after Ramadan, and one month after Ramadan. These patients were assessed using a questionnaire called the GERD health-related quality of life (GERD-HRQL), and antisecretory drugs were prescribed for all patients. This study concluded that Ramadan fasting does not affect the symptoms of GERD in patients taking antisecretory medications such as H2 blockers and PPIs.

Although limited, the currently available literature thus far points to the consensus that fasting with GERD is ultimately safe, and medications are recommended only in certain situations as they have been shown to alleviate GERD symptoms. Several review articles offer specific recommendations for GERD patients, such as dietary composition, eating schedule, medication adjustments, and additional lifestyle modifications. We will further discuss these suggestions in the following section.

Upper GI Bleeding

GERD is the retrograde flow of stomach contents that irritates the esophageal lining, leading to many symptoms of discomfort. Some common symptoms of GERD include retrosternal burning pain, regurgitation, chest pain, and dysphagia. The risk and aggravating factors of GERD include obesity, older age, use of analgesics, smoking, alcohol consumption, intake of caffeinated drinks, and psychological distress [37-39]. As Ramadan fasting is considered a diet, during Ramadan, some of these behaviors are changed for the better, including the cessation of smoking and drinking alcohol. With the reduction of these habits known to worsen GERD symptoms, there may be an alleviation of symptoms during Ramadan [7].

UGIB is bleeding anywhere in the upper GI tract, which consists of everything between the esophagus and the small intestine. UGIB is a severe complication that must be treated immediately since it can be potentially fatal [34]. During Ramadan fasting, specific diet changes are practiced that exclude some of these risk factors, such as the cessation of smoking and consumption of alcohol, which can help to prevent UGIB. Due to the dangerous nature of UGIB, it is essential to gauge whether fasting is suitable for those at risk for UGIB.

A retrospective analysis was performed by Amine et al. [41] on the effect of Ramadan fasting on acute upper GI bleeding (AUGIB). The study focused on 291 patients who were examined for AUGIB through endoscopy during the month of Ramadan and one month before Ramadan, using patient data that spanned from 2001 to 2010. The study showed that there was an increase in patients being treated for AUGIB during Ramadan, as there were 159 patients seen for AUGIB in the month of Ramadan versus 132 in the month before Ramadan. Ramadan fasting had no impact on the patient’s health outcomes, as there were no significant differences between these patients in regard to requiring surgery, rebleeding, the need for therapeutic endoscopy, or mortality rate. Curiously, the most frequent cause of AUGIB during Ramadan was peptic ulcer hemorrhage. The study concluded that prophylactic measures should be taken for those at risk for peptic ulcer disease.

Ozkan et al. [42] conducted a study to evaluate the epidemiological characteristics and health outcomes of patients who presented to the emergency department with acute upper gastrointestinal hemorrhage (AUGIH) during Ramadan in Turkey. This prospective, observational study involved patients aged 16 and older treated at the Department of Emergency Medicine at Erciyes University Medical School. The study found that the number of patients diagnosed with AUGIH during Ramadan was statistically higher than the number of patients diagnosed during other months. The most common diagnosis was a peptic ulcer event. Ozkan et al. [42] concluded by stating that the number of patients being treated for AUGIH during Ramadan was significantly higher than in the non-Ramadan months and that fasting seems to exacerbate the complications and severity of preexisting GI diseases such as peptic ulcer disease and gastritis. Although more patients were being treated for AUGIH during the Ramadan months, there was no significant difference in the outcomes of these patients during Ramadan versus non-Ramadan months.

As observed in the studies conducted by Amine et al. [41] and Ozkan et al. [42], increased presentation of GI bleeding during Ramadan is associated with increased ulcer complications secondary to peptic ulcer disease. Peptic ulcer disease is the leading cause of upper GI hemorrhage, with duodenal ulcers being the most prevalent type [43]. As mentioned above, the available studies on peptic ulcer disease and Ramadan fasting had varying findings regarding gastric ulcer symptom flares and complications, with an increase in surgical interventions required for peptic ulcer perforations in fasting months [35]. Curiously, several studies agreed that the symptom flare of duodenal ulcers worsened during Ramadan, which correlates to duodenal ulcers being the more common source of GI bleeding during Ramadan [41,42].

Although upper GI bleeds were found to be more frequent in those fasting during Ramadan, it is essential to emphasize that the outcome of these patients was not significantly different compared with the non-fasting group [44]. Ramadan fasting can be dangerous for patients with peptic ulcers due to the feared hemorrhage complication. Nevertheless, the literature has concluded that it can be done more safely through the prophylactic use of PPIs [41,44,45]. The studies by Amine et al. [41] and Ozkan et al. [42] showed that the risk of duodenal ulcer hemorrhage without the use of prophylactic PPIs remains increased shortly after Ramadan fasting, which may call for further studies to evaluate duodenal ulcer complications in relation to Ramadan fasting. Although upper GI bleeding has been noted to be more frequent during Ramadan fasting, it is essential to note that certain types of upper GI bleeds may be reduced while fasting, such as esophageal varices [41]. Therefore, future studies may shed light on the incidence and diverse causes of upper GI bleeding to better understand the risks associated with peptic ulcer complications and other preceding conditions that may advance to bleeding.

Liver Diseases

Non-alcoholic fatty liver disease (NAFLD) is a condition in which there is an accumulation of fat in the liver in people who drink little or no alcohol. The cause of NAFLD is unknown. However, risk factors include obesity, gastric bypass surgery, high cholesterol, and type 2 diabetes. Most people with NAFLD do not experience any symptoms. In rare cases, those with NAFLD may experience fatigue, pain, or weight loss. Over time, inflammation (hepatitis) and liver scarring (cirrhosis) can occur. During Ramadan, those who observe fasting follow a strict dietary pattern that involves ceasing food consumption for a predetermined amount of time, which especially significantly alters the liver's metabolic conditions. Restricting food consumption for 14 hours or more depletes the body’s glycogen stores. This shifts the metabolic circuitry to increasing hepatic lipid oxidation, decreasing lipogenesis, and using ketones as the primary energy source [46]. In addition to the potential metabolic benefits of fasting, limiting food consumption during Ramadan fasting can help reduce cholesterol levels, improve blood lipid profiles, and even lead to weight loss. With the reduction in BMI and cholesterol levels and beneficial metabolic changes, Ramadan fasting may potentially improve NAFLD and other liver diseases, such as cirrhosis and steatohepatitis.

A study by Gad et al. [47] was performed on 40 NAFLD patients in an Egyptian hospital during Ramadan 2021 (April-May). The study included both males and females aged 18-65 who had been diagnosed via ultrasound with NAFLD and a controlled attenuation parameter (CAP) score. CAP is a non-invasive assessment of steatosis that measures the increased attenuation of ultrasound waves when traveling through steatotic hepatic tissue. Blood samples were collected before and after patients observed Ramadan fasting. They found that there was a significant decrease in fasting blood glucose, A1C levels, LDL-C, total cholesterol, triglycerides, albumin, total protein, aspartate transaminase (AST), alanine transaminase (ALT), alkaline phosphatase, CAP, and liver stiffness. A significant increase in HDL-C levels in the patients was also observed. Although liver function enzymes decreased after Ramadan, there was no substantial change in bilirubin levels. This study demonstrated that improvements in liver steatosis in patients with NAFLD could occur by fasting during Ramadan.

Derbala et al. [48] conducted a retrospective, controlled observation study using data from the Hamad Liver Transplant Hepatitis Database in Qatar. The study followed 96 patients aged >18 years who were primarily males from the liver transplant clinic between 2008 and 2017. Patients were split into fasting and non-fasting groups with comparisons of lab work, ultrasounds, and liver biopsies. Specifically, the measurements were collected up to four weeks before Ramadan, every two weeks during Ramadan, and up to four weeks after Ramadan. The results indicated no significant differences in the biochemical or hematological indices between the fasting and non-fasting groups. Although there were no notable lab value changes between the fasting and non-fasting groups, the authors questioned if lab values during Ramadan would be different compared to lab values taken outside of Ramadan, as patients’ eating habits may change during the holy month (regardless of fasting state). The authors observed transient changes in both groups. There were significantly higher levels of albumin, total protein, cholesterol, creatinine, hemoglobin, and platelet count before and after Ramadan but not during fasting, suggesting a temporary decrease in these lab values for patients in the study. Of note, no significant change in the model for end-stage liver disease (MELD) score, used to estimate mortality risk, was observed in either group before or after Ramadan. This suggests that while fasting during Ramadan might not lead to changes in lab values that are drastic enough to differ from those of non-fasting patients, it may provide a temporary but beneficial change for liver transplant patients. Due to these results, the authors thought that patients with liver transplants who have stable graft function and do not suffer from cirrhosis could safely observe Ramadan. However, as this was the only study that observed the effects of Ramadan on liver transplant patients at this time, there is room for more research to be conducted, especially in regard to the impact of fasting on the immunosuppressive drugs that many liver transplant patients take.

Elfert et al. [49] conducted a study on 300 patients who were suffering from liver cirrhosis and opted to fast during the month of Ramadan. Approximately 72% of patients completed their fasting. Of these, hepatitis B was the most common cause of liver cirrhosis, followed by hepatitis C and mixed hepatitis B/hepatitis C cirrhosis. The participants were then split into groups based on the child classification (Child A indicating mild cirrhosis, Child B indicating moderate cirrhosis, and Child C indicating severe cirrhosis). There were 105 Child A patients, 91 child B patients, and 20 child C patients. A significant decrease was seen in BMI, glucose, ALT, AST, gamma-glutamyl transferase (GGT), and alkaline phosphatase (ALP) levels after fasting. However, total bilirubin increased after Ramadan, with no significant changes in direct bilirubin. The authors noted several confounding variables that may have influenced the above lab results during Ramadan. Male sex, Child A classification, and the absence of GI bleeding were independent factors that could have influenced the improvement of lab values seen during Ramadan. Older age, diabetes, and the Child C classification were independent predictive factors for increased bilirubin. Overall, it may be that Ramadan fasting can be recommended for patients with mild cirrhosis and a lack of GI bleeding. At the same time, physicians may take more care of elderly patients with more severe cirrhosis or comorbidities such as diabetes.

A study by Mohamed et al. [50] assessed short-term changes in portal blood flow and long-term liver function in cirrhotic patients who fasted during Ramadan. Thirty-eight cirrhotic patients completed the whole month of fasting, with liver function tests, blood work, and the portal vein congestion index via Doppler ultrasonography measured before and after Ramadan. They found a significant increase in portal blood flow after Ramadan compared to before, a feature known to reduce stress on the portacaval anastomosis and possibly lower the risk for dilated vein complications. The study suggested that patients with Child A or B class cirrhosis may fast with close observation, but Child C patients should not fast due to the higher risk of complications. The study ultimately highlighted how patients with less severe cirrhosis might undergo fasting safely with short-term changes in portal blood flow, as these changes were also not linked to any complications or deterioration of liver function.

Liver disease can be a complex pathology, even more so when a physician determines if it is safe for these patients to fast during Ramadan, as each approach depends on the specific patient and their case. Currently, there is room in the literature for further questions to be answered and more elements to be included in future studies. Some examples include larger sample sizes, more prospective studies, patients with comorbidities, patients of different disease stages, and those with liver transplants who are taking immunosuppressants. Within the scope of the effect of Ramadan on patients, there is currently only one study regarding liver transplant patients, and it did not study whether immunosuppressants may also play a role in mediating Ramadan’s effect on patients’ bodies. There is also conflicting evidence on how Ramadan changes bilirubin levels. However, it should be recognized that valuable information may currently guide physicians. One study showed that patients with NAFLD may safely fast during Ramadan while experiencing decreased liver steatosis and stiffness [47]. While the results provide hope that Muslims with NAFLD may observe Ramadan with the added benefit of improvement in their liver steatosis, studies show mixed results. This question should be applied to a larger sample size that excludes comorbidities, as this study included two patients with hypothyroidism. Patients with mild cirrhosis can also partake in the holy month by fasting if they lack GI bleeding. However, this is still a concern for patients who might have mild cirrhosis but are older or have comorbidities. Surprisingly, even liver transplant patients tolerated fasting well without concerning changes in their lab values, with the added benefit of a temporary improvement in their values during Ramadan. While more research is being conducted on this topic, there is promising evidence that liver patients may partake, within reason in their specific case, in Ramadan fasting.

The key findings of the included studies in this section are summarized in Table 1.

**Table 1:** Studies focusing gastrointestinal disorders and Ramadan fasting GI: gastrointestinal, GERD: gastroesophageal reflux disease, IBD: inflammatory bowel disease, PUD: peptic ulcer disease, PPI: proton pump inhibitor, UGIB: upper gastrointestinal bleeding, NAFLD: non-alcoholic fatty liver disease, BMI: body mass index, ALT: alanine transaminase, AST: aspartate transaminase, GGT: gamma-glutamyl transpeptidase, ALP: alkaline phosphatase, LDL-C: low-density lipoprotein cholesterol

Study	Design	Variable under study	Brief of findings
Mardhiyah et al. [7]	Longitudinal study	GERD	GERD symptoms were less severe in those who were fasting during the month of Ramadan versus non-fasting months. GERD symptoms were also less severe in those who fasted during the month of Ramadan versus those who did not fast during the month of Ramadan.
Tavakkoli et al. [28]	Cohort study	IBD	There was no correlation between the number of fasting days and the severity of disease, quality of life, and psychological state of the patient. It appears that Ramadan does not pose serious risk to IBD patients.
El Mountassir et al. [29]	Descriptive retrospective study	Crohn's Disease	94% of the Crohn’s disease patients tolerated fasting and improved clinical symptomatology during the day
Negm et al. [30]	Prospective cohort study	IBD	During Ramadan, patients with ulcerative colitis saw a worsening of clinical parameters especially in older patients.
Ito et al. [31]	Cross-sectional study	PUD	Frequent consumption of margarine, Tsukemono (pickled vegetable) or Cola-beverages may be risk factors for atrophic gastritis whereas foods rich in carotene reduce the risk.
Gokakin et al. [32]	Prospective study	PUD	Duodenal ulcers and duodenitis were found to be more common during Ramadan. Patients with epigastric pain may fast by taking their medications.
Chong [33]	Retrospective study	PUD	There seemed to be no change in the number of patients seen for gastritis, gastric ulcers, or other significant gastric pathologies when comparing other months to Ramadan.
Malik et al. [34]	Prospective study	PUD	Patients who observed Ramadan and followed their drug regimen had improvements in their acute duodenal ulcers and duodenitis. However, patients who fasted and followed their drug regimen did not show improvement in their chronic duodenal ulcers and duodenitis.
Kocakusak [35]	Retrospective study	PUD	Peptic ulcer perforation is significantly higher during Ramadan due to long fasting intervals especially in males.
Ramakrishnan and Salinas [36]	Review article	PUD	Ramadan may exacerbate duodenal ulcers possible due to food consumption actually alleviating ulcer pain which is notably absent during fasting.
Rahimi and Tavakol [40]	Longitudinal study	GERD	Ramadan fasting has no effects on GERD symptoms with those on antisecretory drugs.
Amine et al. [41]	Retrospective Study	UGIB	The most frequent type of UGIB was peptic ulcer hemorrhage and it was seen to be more frequent during Ramadan, however the health outcomes did not change. Prophylactic measures such as PPI use is recommended for those with risk factors for PUD.
Ozkan et al. [42]	Cross sectional study	UGIB	The number of patients experiencing UGIB was greater during the month of Ramadan although the health outcomes of these patients were not worsened.
Sadeghpour et al. [44]	Review article	UGIB	UGIB during Ramadan fasting is more frequent but certain types of UGIB such as esophageal varices are seen to be decreased. Ramadan fasting can cause an increased frequency of UGIB but there is no significant difference in health outcome between those who fasted and those who had not fasted with UGIB.
Abbas [45]	Review article	GERD	Fasting with GERD is safe although empiric use of PPIs are recommended in those who do not follow a GERD friendly diet.
Gad et al. [47]	Prospective study	NAFLD	Patients who fasted during Ramadan had improvements in liver steatosis with lower levels of AST, ALT, A1C, LDL-C, triglycerides, total cholesterol, blood glucose, albumin, and alkaline phosphatase. Higher levels of HDL-C were also observed and there were no significant bilirubin level changes.
Derbala et al. [48]	Retrospective study	Liver transplant patients	Liver transplant patients who fasted showed no significant lab value changes during Ramadan when compared to their non-fasting counterparts. Although lab values do not significantly differ between groups, there is a significant difference in lab values from all groups when comparing them during Ramadan to lab values before/after Ramadan. Specifically, the changes indicate a temporary, beneficial improvement during Ramadan (regardless of fasting state).
Elfert et al. [49]	Prospective study	Liver cirrhosis	Patients with liver cirrhosis (most with hepatitis B as their cause of cirrhosis) had a decrease in their BMI, glucose, ALT, AST, GGT, and ALP levels after fasting. Patients with mild cirrhosis may be able to safely fast during Ramadan.
Mohamed et al. [50]	Prospective study	Liver cirrhosis	Patients with less severe cirrhosis may observe Ramadan fasting safely with short-term changes in portal blood flow, as these changes were also not linked to any complications or deteriorations of liver function. Those with more severe cirrhosis should not fast due to high risk of complications.

Ramadan and recommended lifestyle adjustments: “pre-Ramadan counseling”

Ramadan presents a challenge for physicians when trying to advise Muslim patients. The Quran states, "You who believe, fasting is prescribed for you, as it was prescribed for those before you, so that you may be mindful of God," as an obligatory command for Muslims to fast during the month of Ramadan [2]. This obligation highlights how Muslim patients may insist on fasting depending on their medical condition [51]. For Muslims who fast, a sudden change in dietary habits and lifestyle may necessitate physician attention. Most literature focuses on the challenge of diabetes during Ramadan, understandably, as glycemic control is affected by any change in the diet [52]. Information about pre-Ramadan consultations or their widespread use in primary care clinics is scarce. The following studies provide insight into a path in diet, lifestyle, and approach for physicians who encounter Muslim patients.

General Approach to Muslim Patients During Ramadan

Mahmood et al. [51] gathered data on the experiences of multiple practitioners, patient feedback, and familiarity with Islamic guidance and practice to create a helpful tool for clinicians to facilitate more meaningful discussions regarding Ramadan fasting with Muslim patients. Asking about the patient's history, lifestyle changes, values, previous experience fasting, medication regimen, and assessing fasting length and climate can allow clinicians to stratify patients into very high-, high-, moderate-, or low-risk categories. For very high-risk and high-risk patients, clinicians may counsel the patient regarding concerns with health risks and exacerbation of chronic medical conditions due to Ramadan fasting, thus discouraging these patients from fasting. Moderate- or low-risk patients may fast if lifestyle and medication issues have been addressed. A documented trial before Ramadan can be considered for patients wishing to control their risks before committing to a 30-day fast. A trial fast consists of asking the patient to document their fast (what they ate and the timings) along with identifying individualized risks (any new conditions this Ramadan that changed the severity of conditions, medication changes, and the risk of dehydration or nutritional changes). Three to five "practice fasts" may be recommended in the month preceding Ramadan. Physicians should be adaptable during a trial fast, and patients may be more willing to terminate trial fasts if difficulty occurs. Significance is placed on the shared decision-making between the physician and Muslim patient, which includes discussing safety concerns, medication support, lifestyle, diet, and possible alternatives to provide the patient with the required knowledge to make informed health decisions regarding proceeding with Ramadan fasting.

Similarly, an expert-based guide was developed by Hassanein et al. [52] to provide physicians with a helpful starting point for treating Ramadan patients. Understanding that Muslim patients may still insist on fasting despite their chronic condition is vital, as complications (such as dehydration and hyperglycemia) may arise during the month. Therefore, in-depth discussions that address the risks of fasting are critical in physician-patient interactions. Fasting for Muslims should not present undue suffering for those practicing; the Quran, the Islamic holy book, exempts the sick from the obligation of fasting in the case that fasting leads to detrimental consequences [2]. Additional groups in Islam are also provided exemptions from fasting: pregnant or breastfeeding women, women in their menstrual period or postnatal bleeding, travelers, elderly individuals who cannot handle fasting, mentally disabled individuals, prepubertal children, and those with an acute illness that will worsen with fasting. Physicians should be informed of these exemptions if patients refuse or do not adhere to a necessary treatment to provide proper support and allow the patient to make more informed health decisions. Counseling patients with severe complications from their persistent fasting against medical advice and shared decision-making can aid in achieving safe fasting or understanding their exemption. In the case that patients can continue to fast or gain benefit from fasting in addition to treatment, Islamic scholars from the Islamic Fiqh Council and the Standing Committee for Academic Research and Issuing Fatwas have provided a helpful, specific guide to procedures and drug modifications that do and do not invalidate fasting [3].

Drinking ample fluids, maintaining a healthy diet, structuring exercise between sunset and dawn, altering drug regimens, and adjusting lifestyle modifications to avoid rapid weight changes may provide a helpful basis for initial advice for informing fasting patients. However, patients with varying GI issues may need specific advice for managing clinical symptoms during Ramadan to mitigate risk and improve their condition.

Medication Recommendations

Fasting can also affect the absorption of medications for disorders other than GERD. Given that Muslims are required to refrain from food and liquids and the consumption of oral drugs while fasting, the efficacy of and adherence to drug regimens may be compromised during Ramadan. The typical fasting day can last up to 18 hours, which can be problematic as patients may elect to forgo their medicine, skip doses, or combine multiple doses without medical advice from their physician [53]. As stated in a clinical review by Aadil et al. [54], one or two daily doses are the most common drug regimens used during Ramadan and are typically much easier to follow than medications with multiple doses. However, physicians must keep in mind that Ramadan fasting can alter the circadian rhythm and lead to variations in gastric pH, which may affect the bioavailability of certain drugs [55]. In regards to a two-dose drug regimen, careful consideration must be taken for medications that have a narrow therapeutic index, as the window for consuming food and drugs is limited in Ramadan.

In a study by Daghfous et al. [56], the effects of fasting on theophylline, which has a small therapeutic index, were studied in 12 patients with stable asthma. Patients were given a two-dose regimen for five days during Ramadan when they fasted and one month after the completion of Ramadan when their normal eating pattern resumed. During this regimen, they consumed their medication at times similar to suhoor and iftar, when Muslims are allowed to eat; the first dose was scheduled to be before dawn (3 am), and the second dose was administered at sunset (7 pm). The results showed that two-thirds of patients had nausea and/or abdominal pain with this drug regimen during Ramadan, compared to only one-third of patients during the administration of this regimen after Ramadan. Interestingly, the patients who reported adverse effects during Ramadan had moderately higher plasma theophylline levels than patients who did not report adverse effects. A possible alternative to avoid this complication of high plasma drug levels during fasting would be to utilize a single, slow-release formulation of theophylline for patients who have asthma and observe Ramadan.

Relatedly, the content of meals may also affect the absorption of various medications. Foods high in fiber can impede the absorption of levothyroxine and digoxin, while beverages that increase gastric acidity help aid in the absorption of medications that contain weak acids [54]. Physicians should consider these changes in medication dosing and bioavailability and offer recommendations to improve drug dosing and help patients comply with their treatments during Ramadan fasting.

Dietary Recommendations

When physicians meet Muslim patients during Ramadan, they should keep in mind that the patient's dietary habits are altered compared to non-Ramadan months, especially regarding meal timings and dietary content. In addition to these changes, each Muslim patient may have a cultural background with unique traditional cuisine and dishes with varying nutritional content. With the understanding that there is a scarcity of research on this topic, a data collection study by Shatila et al. [5] aimed to compare the dietary changes of Lebanese adults by utilizing 24-hour dietary recall comparing the month of Ramadan with non-Ramadan months. During Ramadan, they found a decrease in cereal-based products, pasta, eggs, nuts and seeds, milk and dairy products, fats and oils, and an increase in vegetables, dried fruit intake, traditional Arabic pastries, and sugar-sweetened beverages. For physicians, it is important to note the significant dietary changes present in the study: lower intake of fats, cereals, and oils (incredibly saturated fats) and significantly increased dietary fiber, sugar-containing foods, vitamin A, β-carotene, vitamin C, folate, and magnesium. Physicians should consider this as a possible variation in diet during Ramadan when counseling their patients and advising healthier dietary habits during Ramadan, especially for those for whom dietary changes may exacerbate chronic health conditions. Dietary regimens are critical for managing gastrointestinal symptoms. Thus, the intake of certain nutrients during Ramadan fasting may help Muslim patients manage symptoms.

Considering these changes and the fact that many adults participate in Ramadan worldwide, the study emphasizes the importance of providing dietary recommendations that are culturally specific and contextual [5]. The patient's eating habits and nutrient intake should be assessed inside and outside of Ramadan to avoid food-triggering gastrointestinal or other symptoms. It is possible to link dyspepsia, bloating, indigestion, and heartburn to food intake during fasting. Physicians can provide context-sensitive guidance to help patients avoid certain foods and ensure that they consume healthy alternatives when they are fasting. Muslims have cultural backgrounds that include many nourishing foods, and these foods can be encouraged over those identified as triggering GI symptoms [3].

Amini et al. [57] conducted a questionnaire study of 100 participants, analyzing dietary patterns, physical activity, and GI symptoms before Ramadan and during the third week of Ramadan. Muslim adults (≥18 years old) with no medical or religious problems and who could fast during Ramadan were evaluated. The food frequency questionnaire (FFQ) included 168 Iranian foods. As part of the assessment, the Gastrointestinal Symptom Rating Scale (GSRS), which evaluates common GI problems, was used (higher scores indicated more severe symptoms). Before and after three weeks of fasting, participants were assessed for gastrointestinal symptoms. Participants were classified into different dietary patterns according to the most frequently consumed foods before and during Ramadan. Total energy intake did not differ between the groups, but dietary habits changed during Ramadan. Three dominant subgroups among participants before and after fasting were determined based on their most pronounced food groups, as indicated in the questionnaire. The three dominant subgroups before Ramadan are "healthy" (meat, grain, fiber, and nut food groups are more pronounced), "high carbohydrate" (cereal, dairy, cheese, yogurt, and curd), and "high fat" (fast food, cookies, butter, cream, dairy, milk, and ice cream). The three dominant subgroups during Ramadan are "high fat/protein" (meat, fast food, butter, cream, grain, dairy, nut, and cookies), "dairy" (dairy products), and "relatively healthy" (meat, cereal, and fiber).

A comparison was made between each subgroup's dietary patterns and their GSRS scores before and after Ramadan. The findings found that participants in the "high fat" group had significantly reduced constipation and hunger pain but increased diarrhea (possibly due to the laxative effects of fat). The "healthy" group participants had reduced dyspepsia and other GI problems, such as abdominal pain, constipation, and diarrhea. During Ramadan, when dietary patterns had changed, the "high fat/protein" group had significantly reduced dyspepsia and diarrhea prevalence. The "relatively healthy" group had a marginally significant reduction in constipation. Therefore, physicians should consider that patients with symptoms of dyspepsia and diarrhea may benefit from having a consistently healthy diet before and during Ramadan (defined as conspicuous consumption of meat, grain, fiber, and nuts). It is expected that Muslims' eating patterns may change as they enter Ramadan and adjust their fasting regimen, which differs from the rest of the year. If dietary habits change during Ramadan, the study highlights how maintaining a healthy diet (including meat, grains, fiber, and nuts) may help avoid the prevalence of GI symptoms such as dyspepsia and diarrhea. As physicians, having an open conversation with Muslim patients about their diet before and during Ramadan may yield insight into possible causes of their GI symptoms and provide an avenue for improvement through a consistent and healthy diet [58].

GERD-Specific Recommendations

GERD patients who fast during Ramadan can see improvements in their symptoms through positive changes in diet behavior, including the lack of smoking and alcohol consumption [7,45]. However, if they are eating at high volumes before sleeping or ingesting foods or drinks that can exacerbate GERD symptoms, their symptoms might be improved by taking a proton pump inhibitor to safely fast during Ramadan. Dietary alterations, such as avoiding foods and beverages that trigger acid reflux, are also recommended [45].

One recommendation to alleviate GERD occurrences during Ramadan is a Mediterranean diet. Mone et al. [59] conducted a cross-sectional study of adults in Albania, a Muslim-majority country, comparing the rates of GERD among individuals who followed a Mediterranean-based diet (high consumption of fruit and vegetables, olive oil, and fish) in comparison to those who consumed a non-Mediterranean diet (high amounts of red meat, fried meals, sugar, and fast food). They found that a Mediterranean diet decreased the risks of GERD, regardless of other lifestyle factors. Non-Mediterranean diets (such as Western diets) were positively associated with GERD attacks. Similarly, partial dietary modifications are another tool that clinicians can use to help their patients manage GERD. Commonly called elimination diets, the removal of certain foods or beverages previously known to cause GERD flare-ups has been successful in managing some patients afflicted by the condition. In a study by Tosetti et al. [60], patients were asked to modify their diet by eliminating a predetermined list of foods, which included pizza, chocolate, coffee, tomatoes, fried foods, and spicy foods. Upon follow-up, the results showed a reduction in heartburn symptoms in 49% of patients and decreased regurgitation in 44% of patients. To provide clarification, the practice of elimination diets is centered around something other than eliminating random foods and beverages. Instead, foods and beverages that are known to affect digestion and provoke irritation are specifically targeted for removal in GERD patients, such as coffee, alcohol, chocolate, mint, fats, and carbohydrates, which are known to reduce lower esophageal sphincter (LES) tone [45,61]. Due to their long digestion period, fats may be another food to eliminate since they lead to greater acid production and can aggravate GERD [45]. Discomfort may also be relieved by avoiding carbonated beverages, which cause gastric distention and bloating, and spicy foods that irritate the esophageal mucosa [61]. These suggestions become especially relevant regarding eating patterns in the month of Ramadan, when Muslims may consume a larger than normal iftar meal, high amounts of fats and sweets, and practice late-night eating before suhoor [62,63].

Regarding GERD patients who experience symptoms during the fasting day, it would be prudent for physicians to suggest proton-pump inhibitor (PPI) administration 30-60 minutes before the predawn meal (suhoor). Some patients may not have GERD symptoms during fasting but experience postprandial reflux after iftar. Given that PPIs require 3-6 hours to raise gastric pH levels to work effectively, this proves problematic as the patient would have to ingest the PPIs before iftar in order for it to work in time to combat the postprandial reflux. Consuming PPIs before iftar would nullify their fast. A recommended drug alternative that allows the patient to maintain their fast and relieve postprandial reflux more quickly would be alginates or an alginate-antacid combination [64]. These medications can be taken after the iftar meal and have been shown to reduce gastric acidity and reflux more quickly than omeprazole, a PPI [65]. Another way to ease GERD symptoms during Ramadan would be to avoid sleeping shortly after the suhoor meal before dawn. If possible, it is best to wait at least 2 hours before sleeping and to elevate the head of the bed [64,66]. Physicians may suggest these recommendations to GERD patients to actively manage their fasting routines and lower the risk for symptom flares.

The key findings of the included studies are summarized in Table 2.

**Table 2:** Summary of recommendations regarding Ramadan fasting GERD: gastroesophageal reflux disease; PPIs: proton pump inhibitors

Study	Category (diet, medication, behavior, general approach, etc.)	Target patient group	Brief of findings
Aadil and Heber [1]	Medication	All patients	As fasting days can be up to 18 hours long, physicians should consider prescribing medication that follows a 1-2 daily dose schedule as it reduces the need to combine doses after iftar or the risk of patients skipping doses due to fasting.
Al-Marhabi and Wu [6]	Medication	GERD patients	Alginates or an alginate-antacid combination may be considered to replace PPIs during Ramadan due to their more rapid onset action.
Daghfous and Martiny [17]	Medication	All patients	Due to the narrow therapeutic index of some drugs, a slow release formulation may be considered to prevent adverse effects that still occur when patients consume their medications before/after iftar when they can eat.
Mahmood et al. [38]	General Approach	All patients	Physicians should counsel all high-risk patients regarding concerns with health risks and exacerbation of chronic medical conditions; with emphasis on shared decision making.
Kamboj et al. [43]	Diet	GERD patients	Decreased risk of GERD was associated with a Mediterranean-based diet (high consumption of fruit and vegetables, olive oil, and fish) compared to a non-Mediterranean diet (high amounts of red meats, fried meals, sugar, and fast food).
Gad et al. [47]	Diet	GERD patients	Consumption of foods/beverages containing coffee, alcohol, mint, chocolate, excessive fat, spice, and carbonation can aggravate GERD symptoms.
Iraki et al. [55]	Diet	All patients	A high fat/protein diet (defined as conspicuous consumption of meat, grain, fiber, and nuts) during Ramadan was correlated in patients having significantly reduced dyspepsia and diarrhea prevalence.
Mone et al. [59]	Diet	GERD patients	Avoiding a predetermined list of food, which included pizza, chocolate, coffee, tomatoes, fried foods, and spicy foods decreased heartburn and regurgitation symptoms.

## Conclusions

Ramadan fasting has several positive impacts on healthy individuals, from decreasing free fat body mass and reducing systolic blood pressure to decreasing pro-inflammatory cytokines and HbA1c levels. In the gastrointestinal system, Ramadan fasting stabilizes several hormones responsible for appetite control and improves gut microbial composition. Although Ramadan fasting benefits healthy individuals, it may aggravate preexisting conditions such as gastrointestinal diseases. Patients with ulcerative colitis, duodenal ulcers, upper GI bleeding, and moderate-to-severe liver cirrhosis had a higher risk of developing flares and complications associated with Ramadan fasting. Given that Muslims are excused from fasting if it may result in a compromise to health and well-being, physicians should inform their patients of the potential risks associated with fasting and encourage healthier fasting practices such as performing practice fasts to assess tolerance levels, adjusting medications to reduce dosing frequency, avoiding high-fat foods, especially in GERD patients, and promoting a high-protein or high-fiber diet.

While the impacts of Ramadan fasting on prevalent conditions such as diabetes mellitus type 2, hypertension, and hyperlipidemia have been well investigated, there is a need for more research to investigate its associations with further health disorders. Such studies will equip physicians with a deeper understanding of the effects of Ramadan fasting on different systems and conditions, facilitating more definitive discussions between the physician and the patient.
